# MetaboAnalyst 5.0: narrowing the gap between raw spectra and functional insights

**DOI:** 10.1093/nar/gkab382

**Published:** 2021-05-21

**Authors:** Zhiqiang Pang, Jasmine Chong, Guangyan Zhou, David Anderson de Lima Morais, Le Chang, Michel Barrette, Carol Gauthier, Pierre-Étienne Jacques, Shuzhao Li, Jianguo Xia

**Affiliations:** Institute of Parasitology, McGill University, Montreal, Quebec, Canada; Institute of Parasitology, McGill University, Montreal, Quebec, Canada; Institute of Parasitology, McGill University, Montreal, Quebec, Canada; Centre de Calcul Scientifique, Université de Sherbrooke, Sherbrooke, Quebec, Canada; Department of Human Genetics, McGill University, Montreal, Quebec, Canada; Centre de Calcul Scientifique, Université de Sherbrooke, Sherbrooke, Quebec, Canada; Centre de Calcul Scientifique, Université de Sherbrooke, Sherbrooke, Quebec, Canada; Centre de Calcul Scientifique, Université de Sherbrooke, Sherbrooke, Quebec, Canada; Département de Biologie, Université de Sherbrooke, Sherbrooke, Quebec, Canada; The Jackson Laboratory for Genomic Medicine, Farmington, Connecticut, USA; Institute of Parasitology, McGill University, Montreal, Quebec, Canada; Department of Human Genetics, McGill University, Montreal, Quebec, Canada; Department of Animal Science, McGill University, Montreal, Quebec, Canada

## Abstract

Since its first release over a decade ago, the MetaboAnalyst web-based platform has become widely used for comprehensive metabolomics data analysis and interpretation. Here we introduce MetaboAnalyst version 5.0, aiming to narrow the gap from raw data to functional insights for global metabolomics based on high-resolution mass spectrometry (HRMS). Three modules have been developed to help achieve this goal, including: (i) a *LC–MS Spectra Processing* module which offers an easy-to-use pipeline that can perform automated parameter optimization and resumable analysis to significantly lower the barriers to LC-MS1 spectra processing; (ii) a *Functional Analysis* module which expands the previous *MS Peaks to Pathways* module to allow users to intuitively select any peak groups of interest and evaluate their enrichment of potential functions as defined by metabolic pathways and metabolite sets; (iii) a *Functional Meta-Analysis* module to combine multiple global metabolomics datasets obtained under complementary conditions or from similar studies to arrive at comprehensive functional insights. There are many other new functions including weighted joint-pathway analysis, data-driven network analysis, batch effect correction, merging technical replicates, improved compound name matching, etc. The web interface, graphics and underlying codebase have also been refactored to improve performance and user experience. At the end of an analysis session, users can now easily switch to other compatible modules for a more streamlined data analysis. MetaboAnalyst 5.0 is freely available at https://www.metaboanalyst.ca.

## Graphical Abstract

**Graphical Abstract:**
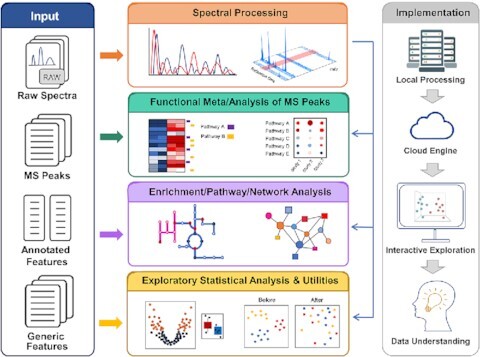
From raw data to statistical and functional insights using MetaboAnalyst 5.0.

## INTRODUCTION

Over the past two decades, metabolomics has contributed significantly to our understanding of metabolism across a broad spectrum of physiological and pathophysiological conditions (1,2). It also plays a leading role in dissecting host-environment interactions (3) and has become an essential component in deep phenotyping for precision medicine (4–7). As with other omics technologies, bioinformatics and analytics go hand-in-hand to enable high-throughput metabolomics data processing, analysis and interpretation. Among a wide array of bioinformatics tools developed for metabolomics (8,9), MetaboAnalyst has been often listed among the popular choices together with XCMS (10) and SIMCA-P (Umetric) etc. The first version (v1.0) of MetaboAnalyst was introduced over a decade ago, focusing on data normalization and statistical analysis (11). Since then, it has undergone continuous growth and co-evolves with metabolomics, encapsulated as milestone releases every three years. The v2.0 expanded to support functional analysis for targeted metabolomics (12). The v3.0 focused on translational biomarker analysis (13) and addressed the performance bottleneck by leveraging cloud computing and modern web technologies (14). The v4.0 further improved on integrative and reproducible analysis (15), and began to support functional interpretation of global metabolomics data (16). With these successive releases, MetaboAnalyst has been steadily gaining and retaining users. According to Google Analytics, this web-based platform has processed over three million jobs submitted from >100 000 users worldwide in the past 12 months alone. For advanced users, the underlying functions have been released as the MetaboAnalystR package to permit more tailored data analysis and batch processing (17–19).

A key limitation of MetaboAnalyst was its limited support for global metabolomics especially with regards to raw data processing. With the consolidation of various protocols and availability of commercial kits developed for targeted metabolomics (20), global metabolomics based on high-resolution mass spectrometry (HRMS) has received growing attention (3,21,22). HRMS instruments such as Orbitrap or time-of-flight (TOF) systems can simultaneously measure a vast number of endogenous and exogenous compounds in a biological sample, providing unique information on an individual's metabolic phenotype, environmental exposures and associated biological responses. However, HRMS data processing is currently a labor-intensive task involving significant user input, as many parameters need to be empirically tuned in order to obtain satisfying results (23,24). To democratize the power of HRMS to researchers beyond a few expert groups, we need to overcome two major hurdles - developing a self-tuning algorithm to enable automated parameter optimization and implementing a high-performance computational platform to deal with the big data challenges associated with raw data processing.

For most researchers, the peak tables obtained from raw data processing are not interpretable. The conventional approaches such as pathway or enrichment analysis require peaks to be identified first to gain functional insights (25). Therefore, it is necessary to enhance the support for functional analysis directly based on peak tables. With the availability of public metabolomics repositories (26,27), there is a growing interest in data mining and meta-analysis. However, the heterogeneity of global metabolomics datasets due to differences in analytical platforms and data processing parameters has posed significant challenges for this purpose. Addressing this need will greatly improve the value of global metabolomics datasets. Finally, improved support for lipidomics data, better integration with other ‘omics’ data, batch-effect correction, *etc*. have been among the common requests from the MetaboAnalyst users.

Here, we introduce MetaboAnalyst version 5.0, which represents our three years of effort to narrow the gap between raw HRMS spectra and functional insights since the release of the version 4.0. The key features of MetaboAnalyst v5.0 include:

1. A new module to support high-throughput, self-optimized LC-MS1 spectral processing.
2. A new module to allow meta-analysis of multiple global metabolomics datasets.
3. A weighted joint pathway analysis module for multi-omics integration, and a new function for data-driven network analysis (28).
4. Significantly updated and expanded underlying knowledge bases (species-specific pathway libraries and metabolite sets) for comprehensive functional analysis of both targeted and untargeted metabolomics.
5. Completely upgraded interactive graphics, refactored underlying codebase for improved performance and streamlined data analysis across compatible modules.
6. Other new features including support for mzTab 2.0-M (29) input format and importing data from the Metabolomics Workbench (26), as well as utility functions for automated batch correction and merging technical replicates.

The MetaboAnalyst v5.0 is freely available at https://www.metaboanalyst.ca. To accommodate computational demand, we have also set up two mirror sites hosted on high-performance computers dedicated for raw data processing. We have updated frequently asked questions (FAQs) and added seven new tutorials, which are easily accessible from the home page. The key features of MetaboAnalyst 5.0 are described below.

### Overview of Metaboanalyst 5.0 workflow

**Figure 1.**
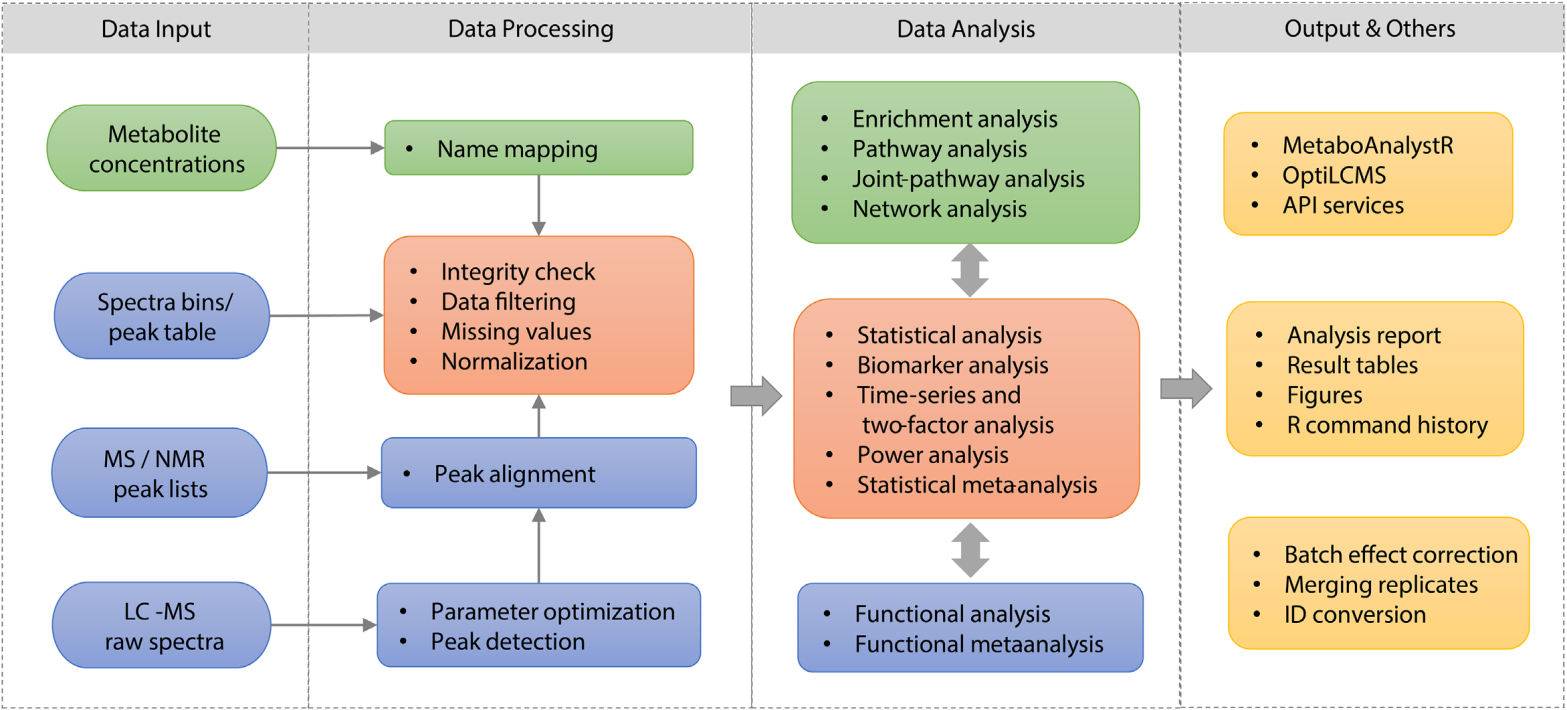
Overview of MetaboAnalyst v5.0 workflows. Steps for targeted metabolomics are indicated by boxes in green, steps for untargeted metabolomics are in blue, and those in orange can be used for both. Experienced users can use various utility functions or install the corresponding R packages (yellow boxes) to perform analysis beyond those pre-defined regular workflows.

In addition to supporting raw data processing for MS-based global metabolomics, MetaboAnalyst version 5.0 harmonizes workflows for both targeted and untargeted data analysis. As summarized in Figure 1, after proper data processing, all main inputs can be handled consistently within the framework of statistical analysis, functional analysis and meta-analysis with coherent interface design and navigation support. Altogether, these updates allow users to easily perform their analytical workflow and focus more on understanding their own data rather than how to operate the tool.

### Raw data processing

Over the past 15 years, XCMS and MZmine have evolved into the two most popular, open-source tools for HRMS raw data processing (30,31). Both now use the *CentWave* algorithm for chromatographic peak detection (32). However, multiple parameters often need to be specified beforehand in order to obtain good results, which has caused challenges for its practical applications even for an experienced analyst. The XCMS Online platform has partially addressed the issue by offering several pre-optimized platform-specific parameters (10,33). However, more refined parameter optimization is usually necessary, because chromatography can vary greatly between laboratories, and the spectral data are influenced by sample preparation and many configurations or conditions of mass spectrometers.

We have recently developed a self-tuning parameter optimization method for XCMS-based HRMS spectra processing and benchmarked its performance against other well-established approaches (17). The algorithm was initially developed as a component in MetaboAnalystR 3.0. Based on user feedback, we recently extracted and optimized the algorithm as an independent R package (OptiLCMS, https://github.com/xia-lab/OptiLCMS) to be embedded in other pipelines. This pipeline is designed to automatically identify the optimal parameters for a user-provided dataset in an efficient manner. Briefly, the ‘automated optimization’ pipeline will select multiple regions of interest (ROIs) across the whole spectra as the training spectra. Then, a design-of-experiment (DoE) optimization will be executed to find out the combination of parameters with the most well-behaved peak shape and stable peak groups to be applied to whole dataset for peak detection. MetaboAnalyst v5.0 offers this pipeline via its user-friendly web interface to support both automated and manual parameters optimization to accommodate both regular and expert users. We have also developed a resumable workflow to accelerate data re-analysis after parameter update. To accommodate a wide range of spectral data qualities, we recently implemented a function to detect and exclude common background noises and experimental contaminants during the parameter optimization stage. Specifically, all *m*/*z* centroids from the whole spectrum will be extracted first and those m/z features appearing consecutively across half of the entire chromatogram will be excluded for parameters’ optimization. Following peak detection and alignment, the annotation of adducts and isotopes is based on the CAMERA R package (34). The pipeline is now available as the new *LC–MS Spectral Processing* module in MetaboAnalyst v5.0.

**Figure 2.**
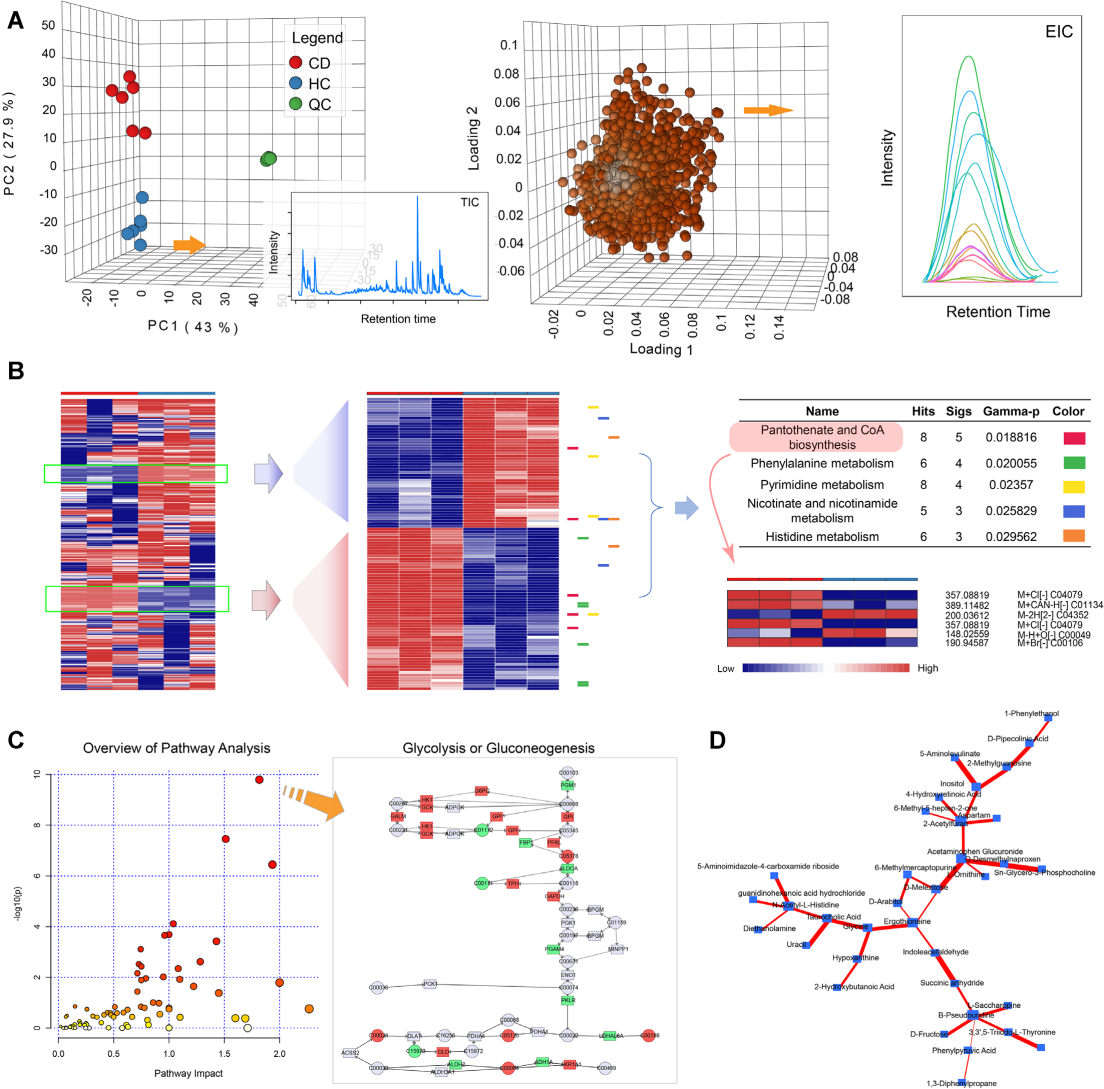
Example outputs from several new features of MetaboAnalyst v5.0. (**A**) Interactive PCA scores and loadings plots generated from the Raw Data Processing module. Users can click any samples or features to view their spectra; (**B**) Enrichment analysis of patterns detected in a peak table from the Functional Analysis module. Users can drag-select any patterns and test their enriched functions for further exploratory analysis; (**C**) An example output from the Joint-Pathway Analysis module. Users can click any data points to view the underlying pathways; (**D**) An example output from the DSPC network analysis.

Users can upload up to 200 data files in the supported open data formats (mzML, mzXML, netCDF or mzData). Since raw data processing is a time-consuming process, users can create and save a bookmark link after job submission. The link is used to check their job status and to retrieve the result. Alternatively, users can freely create accounts using their emails for better data management and communication. Registered users can create up to 10 projects, revisit or re-analyze their data later. When raw spectral processing is complete, users can visually inspect their results in an interactive 3D PCA plot (Figure 2A), as well as view total ion chromatogram (TIC) plots, base peak intensity (BPI) plots, retention time correction results, etc. Furthermore, users can click any feature of interest to view its corresponding extracted ion chromatogram (EIC) plot. From the Results Download page, users can download all the processed data and peaks tables or start a new journey to other compatible modules.

### Functional analysis of MS peaks

It is now possible to directly translate a HRMS peak table into biological insights after raw data processing. MetaboAnalyst v4.0 first implemented the ‘MS Peaks to Pathways’ module based on the *mummichog* algorithm (16). Briefly, the algorithm first performs putative annotation of MS peaks considering different adducts and ion modes. These putative compounds are then mapped onto user selected pathway libraries for pathway activity prediction. The previous version (*mummichog* version 1) only considered the m/z dimension. In MetaboAnalyst v5.0, we have upgraded the algorithm to version 2 by integrating both *m*/*z* and retention time dimensions to formulate empirical compounds, thereby further improving the accuracy of functional interpretation (17). Both versions of the *mummichog* algorithm are now available in MetaboAnalyst v5.0. The new interface also allows advanced users to customize the default adduct lists and currency metabolites - ubiquitous compounds such as water, oxygen, carbon dioxide, *etc*. (35).

The typical application of the *mummichog* algorithm is to predict pathway activities based on a list of MS peaks ranked based on t-tests. The concept can be generalized to test enrichment of any predefined function (i.e. metabolite sets) in any peak groups of interest (i.e. a cluster of similar peaks instead of significant peaks). Herein, we have implemented an interactive heatmap to allow users to perform functional analysis on any manually selected region of interest. In this case, the uploaded peak intensity table will be first visualized as an interactive heatmap (36). Users can perform cluster analysis with different methods, and then specify (via drag-select) one or more patterns of interest. The *mummichog* will be applied to predict enriched functions for the selected peaks. From the result, users can click any function name (i.e. pathway or metabolite set) to see the corresponding features annotated beside the heatmap (Figure 2B).

### Meta-analysis of global metabolomics data

It is notoriously challenging to integrate untargeted metabolomics data across different studies, because different extraction methods, chromatographic conditions and mass spectrometry platforms all lead to heterogeneity of HRMS data. This issue has precluded the use of untargeted metabolomics datasets for large-scale meta-analysis using conventional statistical methods (37). To address this gap, we have developed a new module to enable researchers to perform functional meta-analysis of global metabolomics datasets.

Users can submit multiple peak intensity tables obtained from the same (or very similar) diseases or phenotypes of interest. The meta-analysis can be performed by pathway-level integration or by pooling peaks. If the studies are independent of each other (i.e. different samples) but interrogate more or less the same pathways, the integration should be performed at the pathway level. In this case, the pathway analysis will be first performed on each dataset and the final significant pathways will be identified based on the integrated p-values. The results can be visually explored in an interactive Venn diagram. In contrast, the peak pooling strategy aims to improve the metabolome coverage by combining complementary information obtained under different experimental conditions (i.e. compound extractions, chromatographic conditions, ion modes, *etc*.) from the same set of samples using the same or very similar MS instruments. The results can be visually explored in a KEGG metabolic network. The utility of the pathway-level integration has been demonstrated in our recent meta-analysis of COVID-19 global metabolomics datasets (38).

### Multi-omics integrative analysis

Integrating data from different omics layers can provide greater resolutions to reveal mechanistic insights as compared to using a single omics profile. Multi-omics integration can be either data-driven based on multivariate statistics (39) or knowledge-driven based on known pathways or molecular interaction networks (40). In practice, both data-driven and knowledge-driven approaches can be further integrated to maximize information gain (41,42).

Integrated pathway analysis of genes and metabolites was first launched as the ‘Joint Pathway Analysis’ module in MetaboAnalyst v3.0 by directly concatenating genes and metabolites into a single query (i.e. combining queries) followed by over-representation analysis. However, the results are often dominated by transcriptomics data which tends to yield many more significant features than metabolomics. To address this issue, we have added three new options for combining *P*-values from different tests of the same hypothesis (43), including one unweighted (Fisher's method) and two weighted approaches (Stouffer's *Z*-score method). The weights are the proportions of genes or metabolites within the combined universe (overall) or within individual pathways (pathway-level) (Figure 2C). Four types of pathway libraries are provided. Users can choose metabolic pathways or all pathways (including signaling pathways) for integrated analysis. The other two types - metabolic pathways (metabolite only) and all pathways (gene only) allow users to perform pathway analysis for individual omics data.

The integration of transcriptomics and metabolomics data can also be explored using the Network Analysis module. The knowledge-based network integration has been established since MetaboAnalyst v4.0. However, such an approach excludes the high volume of unannotated MS features detected by HRMS. We added the support for data-driven network analysis by implementing the well-established debiased sparse partial correlation (DSPC) algorithm (28). Briefly, networks are created using a graphical LASSO model to compute the partial correlation coefficients and *P*-values for every pair of features in the dataset (44). The result can be visually explored as an interactive network with node size corresponding to node degrees and edge thickness based on the correlations between two connecting nodes (Figure 2D). The DSPC network is applicable to both targeted and global metabolomics and can be accessed from either Network Analysis or Statistical Analysis module.

### Extended knowledge bases

The underlying knowledge bases within MetaboAnalyst have undergone significant updates to ensure that users’ inputs can be identified correctly and accurately. These improvements are summarized as below.

#### Compound database

The compound databases, used by the Enrichment, Pathway, Joint-Pathway and Network Analysis modules have been enhanced by updating chemical identifiers from HMDB (45), KEGG (46), PubChem (47) and ChEBI (48). We have also expanded the database by including an additional 197 854 lipids from RefMet (49) and LIPID MAPS (50).

#### Metabolite sets

Metabolite sets, which are groups of metabolites with shared biological functions or collective behaviors, regulations or structures, are the backbone of the Enrichment Analysis module. To enhance these sets, we have added 44 metabolite sets related to disease signatures found in fecal samples, as well as 1571 metabolite sets identified by RefMet (49) and LIPID MAPS (50). These metabolite sets have also been transformed into appropriate libraries for Functional Analysis module for global metabolomics. Users can now identify perturbations in organism-specific metabolic pathways or metabolite sets from raw spectra or peak lists.

#### Pathway libraries

Pathway libraries are used by the Pathway, Joint Pathway and Network Analysis Modules. All KEGG pathways libraries have been updated with the latest information from KEGG using their API (46). Additionally, we have added five new species (*Plasmodium vivax*, *Chlorella variabilis*, *Klebsiella pneumoniae*, *Klebsiella variicola* and *Streptococcus pyogenes*) based upon users’ requests. The global KEGG metabolic network has also been updated to the latest version for the Network Analysis and Functional Analysis modules.

### Other features

#### Enhanced visualizations

We have systematically updated the interactive plots across several modules (Enrichment, Pathway, Statistical and Biomarker Meta-Analysis), including synchronized 3D scatter plots for Principal Component Analysis (PCA) and Partial Least Squares - Discriminant Analysis (PLS-DA), as well as interactive volcano plots, bar plots, pie charts, and 2D scatter plots using the powerful Chart.js library (https://www.chartjs.org/). Furthermore, we have enhanced several publication quality graphics in the Statistical Analysis module such as box plots, K-means and self-organizing map (SOM) overview plots. Finally, users can now customize the colors and shapes of groups or samples in many important images.

#### Improved compound name matching

To provide better support for lipidomics data, we have implemented a smart name matching algorithm to improve the mapping from a user uploaded list or table of lipid names with our internal compound database. This algorithm considers common lipid abbreviations used by the LIPID MAPS classification system (51) as well as variations in punctuation marks used by different companies or databases. Compound synonyms for all metabolites in our internal compound database have been complemented from HMDB, PubChem and LIPID MAPS. This algorithm is used in all compatible modules within MetaboAnalyst v5.0.

#### Automated batch effect correction

The utility function for batch effect and signal drift correction has been updated with eight algorithms-*EigenMS* (52), *QC-RLSC* (53), *ANCOVA* (54), *RUV-random* (55), *RUV2* (56), *RUVseq* (57), *NOMIS* (58) and *CCMS* (59) for correction based on either the data itself, QC samples or internal standards. The highlight for this update is the ‘automated’ design that can automatically identify and perform the optimal correction for the results (17). Users can upload the batches individually or as a merged table with all data together. All applicable correction methods will be executed, and the best results indicted by the distance among the batches will be returned.

#### Merging technical replicates

Technical replicates improve the stability and reproducibility of global metabolomics (43). However, averaging signals across the replicates may not be the best approach. We developed a new utility function to handle technical replicates in MetaboAnalyst v5.0. For a certain feature with multiple replicates, if the missing proportion in the replicates is over 1/3, the coefficient of variation (CV) of the feature in these replicates will be evaluated (60). If the CV is over 1.0, this feature will be considered ‘*highly variant*’ with an assigned value of zero. A kernel density estimator is also available for users to smooth their data.

#### Supporting new input formats

The mzTab-M is a standard quantitative metabolomics data format (29). The latest version of this data format (version 2.0) is now supported by MetaboAnalyst v5.0. The Metabolomics Workbench is one of the most popular data repositories for metabolomics (26). We have added support to allow users to easily perform analyses on published datasets deposited in the Metabolomics Workbench. Users simply need to input the study ID of their preferred dataset. MetaboAnalyst will then retrieve the deposited data table for further statistics, functional enrichment, biomarker or network analysis.

#### Streamlined data analysis

A major effort in v5.0 is to refactor the underlying software architecture to enhance the modular structure and to improve the interoperability among different modules. With this update, modules can be developed and tested more independently, and users can now switch to other compatible modules at the end of each analysis, therefore creating their own custom pipelines.

### Implementation

The web component of MetaboAnalyst v5.0 is implemented using the PrimeFaces framework (https://www.primefaces.org/). The core functions and graphics are executed using R (v4.0.2) and are freely available from the GitHub repositories as MetaboAnalystR (https://github.com/xia-lab/MetaboAnalystR) and OptiLCMS (https://github.com/xia-lab/OptiLCMS). The main site of MetaboAnalyst is hosted on a Google Cloud Server (with 64GB RAM and eight virtual CPUs with 2.6 GHz for each) for general data analysis except for the raw data processing module. To accommodate the computing demand for raw data processing, we have set up two additional computing nodes located at the McGill Data Center and Compute Canada through a collaboration with the GenAP project (genap.ca), respectively, with 1TB RAM and 50TB of storage in total. These two websites are linked with the main site. Users can choose whether to register an account to manage their jobs. A maximum of 40GB data volume is allocated for each project (at most 10 projects for each registered user). The job submission and scheduling are based on the Simple Linux Utility for Resource Management (SLURM) system. During the upgrade to v5.0, we have made every possible effort to ensure backward compatibility with v4.0. For those who still need to access MetaboAnalyst v4.0, we have made it available as a Docker image (https://github.com/xia-lab/MetaboAnalyst_Docker).

### Comparison with other web-based tools

Several web-based tools are available for metabolomics data analysis. Here we compared MetaboAnalyst v5.0 with these tools as well as the previous two versions (v4.0 and v3.0). The main features and characteristics of different tools are summarized in Table 1. Compared to the previous versions, the v5.0 has significantly enhanced many features and is distinctive in raw data processing and functional analysis for global metabolomics. Among other web-based tools, XCMS Online is well-known for raw data processing (10). MetaboAnalyst compares favorably with XCMS Online in several aspects including optimized raw data processing and downstream statistical and functional analysis, while XCMS Online excels in compound annotations based on the METLIN database (61). Among the remaining tools, Workflow4Metabolomics (W4M) (62) is a Galaxy-based workflow which uses the XCMS package for raw LC–MS data processing. The default workflow does not include a parameter optimization step, although experienced users can customize the pipeline to include IPO (63). In addition, W4M supports other types of raw metabolomics data including GC–MS and NMR. The two other tools - 3Omics (64) and NOREVA (65) mainly focus on metabolomics data integration and normalization, respectively. MetaboAnalyst v5.0 remains the most comprehensive web-based platform that enables user-friendly and streamlined metabolomics data analysis and interpretation.

**Table 1.** Comparison of MetaboAnalyst (versions 3.0–5.0) with other web-based tools. Symbols used for feature evaluations with ‘√’ for present, ‘-’ for absent, and ‘+’ for a more quantitative assessment (more ‘+’ indicate better support)

	MetaboAnalyst						
Tools name	5.0	4.0	3.0	XCMS Online	W4M	3Omics	NOREVA
**Raw spectral processing**							
Parameter optimization	+++	−	−	+	++	−	−
Supported algorithms	+++	−	−	++	++	−	−
Resumable analysis	+++	−	−	−	+	−	−
Compound annotation	+	+	−	+++	++	−	−
**Statistical analysis**							
Univariate	+++	++	++	+	+	−	+
Multivariate	+++	++	+	+	+++	−	++
Clustering	+++	+++	++	+	+	−	−
Power analysis	√	√	√	−	−	−	−
Time-series analysis	√	√	√	−	−	−	√
Biomarker analysis	√	√	√	−	−	−	−
Biomarker meta-analysis	√	√	−	−	−	−	−
**Functional analysis**							
Function analysis (MS peaks)	+++	++	−	++	−	−	−
Enrichment analysis (compounds)	+++	+	+	−	−	++	−
Pathway analysis	+++	++	+	−	−	++	−
Functional meta-analysis	+++	−	−	++	−	−	−
**Integrative analysis**							
Unbiased joint pathway	+++	+	+	−	−	+++	−
Knowledge-based Network	++	++	−	−	−	++	−
Correlation-based Network	++	−	−	−	−	−	−
**Other features**							
Data normalization	++	+	+	−	+	−	+++
Missing value estimation	√	√	√	−	−	−	√
Technical Replicates Merging	√	−	−	−	−	−	−

• XCMS online: https://xcmsonline.scripps.edu/.

• Workflow4Metabolomics (W4M): https://workflow4metabolomics.usegalaxy.fr/.

• 3Omics: https://3omics.cmdm.tw/.

• NOREVA: http://idrblab.cn/noreva/.

## CONCLUSION

We have implemented a fully automated workflow to perform optimized peak detection, alignment and annotation tasks for LC–MS1 data generated in global metabolomics. The workflow can be easily accessed via the user-friendly web interface of MetaboAnalyst v5.0 or can be installed locally as an R package. We have also enhanced functional analysis by allowing biological interpretation directly from any peak groups or patterns of interest. The functional meta-analysis module further enables users to integrate heterogeneous global metabolomics datasets for improved understanding. We have also updated the compound databases and pathway libraries to enable comprehensive functional analysis for a wide range of species. During the process, we have consolidated the majority of modules in terms of interface, graphics and code architecture to improve user experience and performance. Overall, MetaboAnalyst v5.0 has addressed important gaps in the current metabolomics data processing and analysis pipeline. In the future, we aim to support more vendor data formats for raw spectral processing and to support spectral deconvolution based on tandem MS data.
